# Supplementary material for: Novel Driver Strength Index highlights important cancer genes in TCGA PanCanAtlas patients
Source: PeerJ. 2022 Aug 11;10:e13860. doi: 10.7717/peerj.13860 (PMC9375969; doi:10.7717/peerj.13860)
Supplement: Supplemental Information 2 [file peerj-10-13860-s002.zip › COHORTS/genes plots/age/2021_8_16_14_9_distribution_cohorts_females__25.pdf]

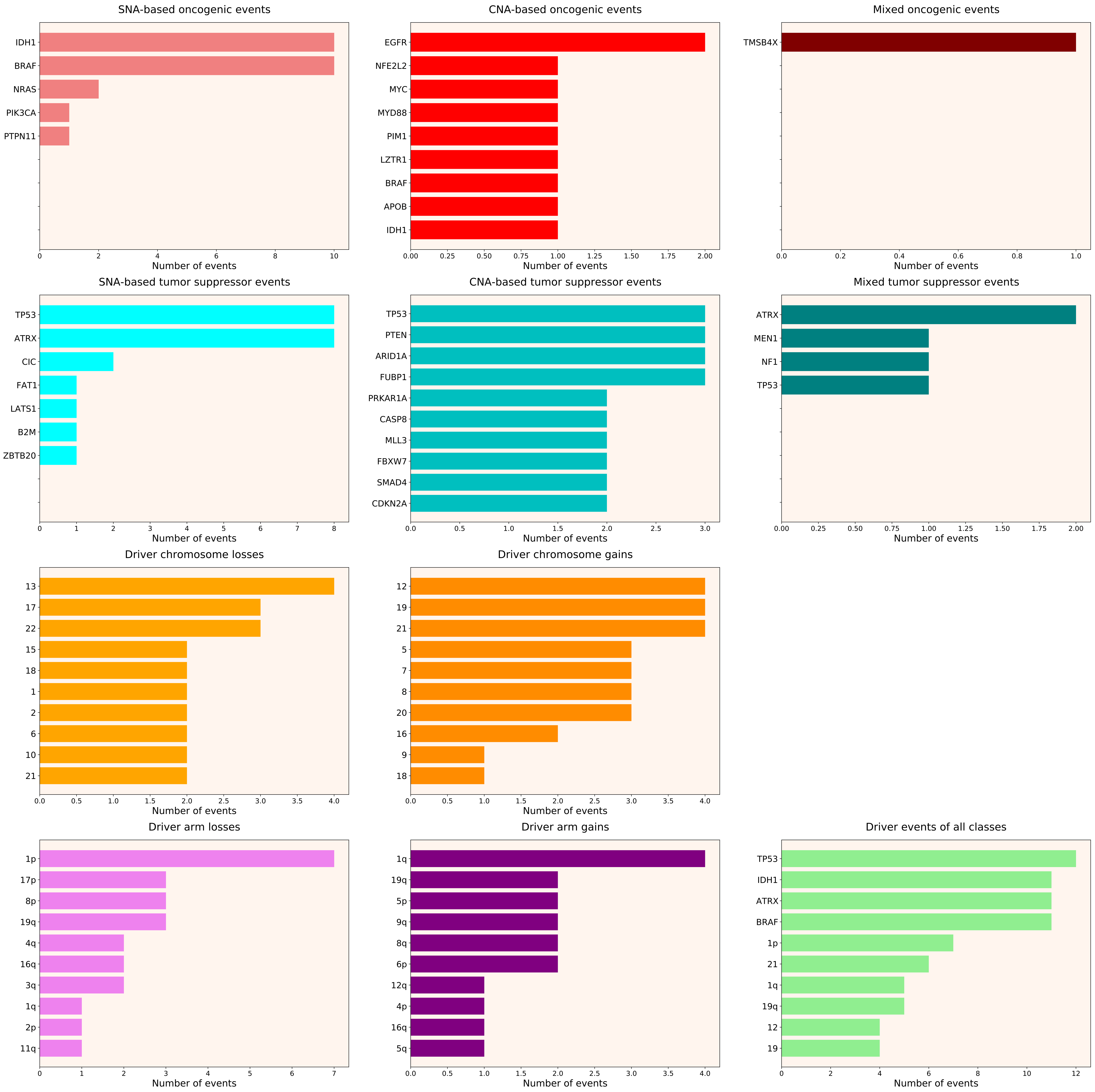

SNA-based oncogenic events
IDH1
BRAF
NRAS
PIK3CA
PTPN11
Number of events
CNA-based oncogenic events
EGFR
NFE2L2
MYC
MYD88
PIM1
LZTR1
BRAF
APOB
IDH1
Number of events
Mixed oncogenic events
TMSB4X
Number of events
SNA-based tumor suppressor events
TP53
ATRX
CIC
FAT1
LATS1
B2M
ZBTB20
Number of events
CNA-based tumor suppressor events
TP53
PTEN
ARID1A
FUBP1
PRKAR1A
CASP8
MLL3
FBXW7
SMAD4
CDKN2A
Number of events
Mixed tumor suppressor events
ATRX
MEN1
NF1
TP53
Number of events
Driver chromosome losses
13
17
22
15
18
1
2
6
10
21
Number of events
Driver chromosome gains
12
19
21
5
7
8
20
16
9
18
Number of events
Driver arm losses
1p
17p
8p
19q
4q
16q
3q
1q
2p
11q
Number of events
Driver arm gains
1q
19q
5p
9q
8q
6p
12q
4p
16q
5q
Number of events
Driver events of all classes
TP53
IDH1
ATRX
BRAF
1p
21
1q
19q
12
19
Number of events
